# Influence of clinical risk factors for preterm premature rupture of membranes (PPROM) on the elastic strength of fetal membranes at term: A prospective study

**DOI:** 10.1371/journal.pone.0312760

**Published:** 2024-12-03

**Authors:** Amaury Robin, Nicolas Tessier Doyen, Sami Ben Rhaiem, Nancy Valette, Véronique Fermeaux, Pierre-Marie Preux, Sophie Martinez, Jean-Luc Eyraud, Chahrazed El Hamel, Didier Riethmuller, Perrine Coste Mazeau

**Affiliations:** 1 Gynecology and Obstetrics Department, CHRU Limoges, Limoges, 87000, France; 2 Institut de Recherche sur les Céramiques, University Limoges, UMR CNRS 7315, Limoges, 87068, France; 3 Anatomopathology Department, CHRU Limoges, Limoges, 87000, France; 4 CDCR, Clinical and Research Data Center, CHRU Limoges, Limoges, 87000, France; 5 Pediatric Department, Mother and Child Biobank (CB-HME), Hôpital de la Mère et de l’Enfant, CHU Limoges, Limoges, 87000, France; 6 Gynecology and Obstetrics Department, CHRU Grenoble, Grenoble, 38000, France; 7 University of Limoges, Inserm, CHU Limoges, RESINFIT, U 1092, Limoges, F-87000, France; Kasr Alainy Medical School, Cairo University, EGYPT

## Abstract

**Introduction:**

Premature rupture of membranes (PROM) before 37 weeks of gestation is a common obstetrical event, whose pathophysiology is still poorly understood. Our objective was to study the mechanical strength of fetal membranes in women with a clinical risk factor for preterm premature rupture of membranes (PPROM).

**Methods:**

We included, in a prospective, descriptive, single-center study, patients scheduled for cesarean section at term (≥ 37 weeks of gestation). For each patient, we performed uniaxial tensile tests on fetal membranes with a universal testing machine equipped with a force sensor (EZ20^®^, Lloyds), allowing the recording of an applied force/time curve. We collected maximum force (Fmax), maximum stress (σMax), and Young’s modulus of elasticity. The thickness of each membrane sample was also measured. We compared the values obtained according to certain clinical risk factors for PPROM such as age, body mass index, gravidity, parity, a history of PPROM or preterm birth, smoking, gestational diabetes, geographic origin, and socioeconomic level.

**Results:**

We analyzed 31 patients and found no association between the studied risk factors and σMax. Fmax was lower in primiparous patients (p = 0.02) but increased with patient parity (p = 0.005). Gestational diabetes was associated with a higher Fmax (p = 0.033) and sub-Saharan geographical origin with a greater thickness (p = 0.0043). As membrane thickness increased, σMax (p = 0.009) and Young’s modulus decreased (p = 0.037).

**Conclusion:**

Primiparous patients have lower membrane mechanical strength than patients who have had one or more deliveries. Mechanically, the thicker membranes are less rigid and less resistant.

## Introduction

Premature rupture of membranes (PROM) before 37 weeks of gestation (WG) occurs in 2 to 4% of pregnancies and is responsible for one-third of premature deliveries and thus for 20% of perinatal mortality [1]. When preterm premature rupture of membranes (PPROM) occurs very early (<24 WG), it can lead to prolonged anhydramnios, which can be responsible for severe fetal pulmonary hypoplasia, but also to arthrogryposis.

Several studies have been performed, often retrospectively, on the clinical factors associated with PPROM, but the pathophysiological link cannot always be established. Demographic and sociobehavioral factors (smoking, African ethnicity…) [2–5], maternal characteristics (age, high or low body mass index (BMI)) [2,3,6–10], or obstetric factors (nulliparity, multiple pregnancies, shorter interpregnancy interval, polyhydramnios or gestational diabetes…) [10–14] have been described to be associated with an increased risk of PPROM. A history of PPROM or prematurity, are predominant risk factors [11,15–16] which may be caused by the existence of cervical incompetence or structural abnormalities of the uterine wall [17] or, according to Assefa et al. delayed treatment of reproductive tract infections [18].

Fetal membranes are formed by the juxtaposition of two layers of fetal origin: the amnion and the chorion, and a third layer of maternal origin strongly adherent to the chorion, the maternal decidua. These elements form a choriodecidual membrane composed of several superimposed cell layers which form, with the placenta, a true fetomaternal interface, which constitutes both a passive, physical barrier and an active, immunological barrier, which fights infection. It is also a rich site for the emission of autocrine and paracrine signals which play an important role in both the maintenance of pregnancy and in labor leading to birth [1].

The etiological mechanisms leading to rupture of the membranes, physiological as in rupture at term with labor or pathological with PPROM, are still poorly understood. Although infection and inflammation are the two main causes of PPROM, mechanical stress on fetal membranes could also be at the origin of PPROM [19,20] and this has been explored in previous studies [21–24]. Thus, our work focused on the study of certain etiological factors described as associated with a risk of PPROM. To understand the pathogenesis of these elements, we performed an experimental study of the mechanical elastic resistance of fetal membranes and a pathological study of membrane tissue thickness outside an infectious context.

## Material and methods

### Study design

We performed a prospective, descriptive, single-center study in our type 3 maternity unit (2500 deliveries per year) in the Gynecology and Obstetrics Unit of the Limoges Regional University Hospital, in France, between January 2022 (first patient included on January 6, 2022) and June 2022 (last patient included on June 7, 2022).

### Study population

For 6 months, we consecutively included patients scheduled for cesarean section (fetus in breech or transverse presentation, uterus with several previous caesarean sections…) who were ≥18 years old, with a singleton pregnancy of gestational age ≥37 WG. We excluded patients who had PPROM, PROM or went into labor before cesarean delivery, had infectious disease (human immunodeficiency virus, hepatitis C virus, hepatitis B virus), or obstetrical complications (except for gestational diabetes). In our center, we perform scheduled cesarean sections at term between 37 and around 39 WG, and delivery of patients with gestational diabetes is preferably scheduled around 39 WG.

### Procedures

For each patient who signed the consent form, the cesarean delivery was performed under the usual conditions, with only a "delicate" manual delivery required of the operator. The placenta was then stored at 4°C until it was cut up the same day. On the day of cesarean section, the placenta was included in the CB-HME collection (declared to the Ministry of Education and Research (DC-2011-1264) and approved by the ethics committee CPP Sud-Ouest Outremer IV, Limoges, France) and was then anonymized. A section of the membranes overlying the cervix (ZAM = zone of altered morphology), identified and marked during surgery, was sent at room temperature during the day to the laboratory for sampling and mechanical testing. A membrane section further away from the ZAM was then made and sent to the pathology laboratory for fixation and storage. The remaining placental mass was discarded without analysis. All experiments were performed by two persons.

For each patient we collected the following variables: maternal age at the time of inclusion, gestational age and parity, BMI, smoking status during pregnancy, history of prematurity or PPROM, presence of gestational diabetes, geographical origin (patients classified into 2 groups: patients from Europe and North Africa, and patients from sub-Saharan Africa or the Caribbean) and socioeconomic level (low/medium/high).

### Mechanical tests

**Fig 1:**
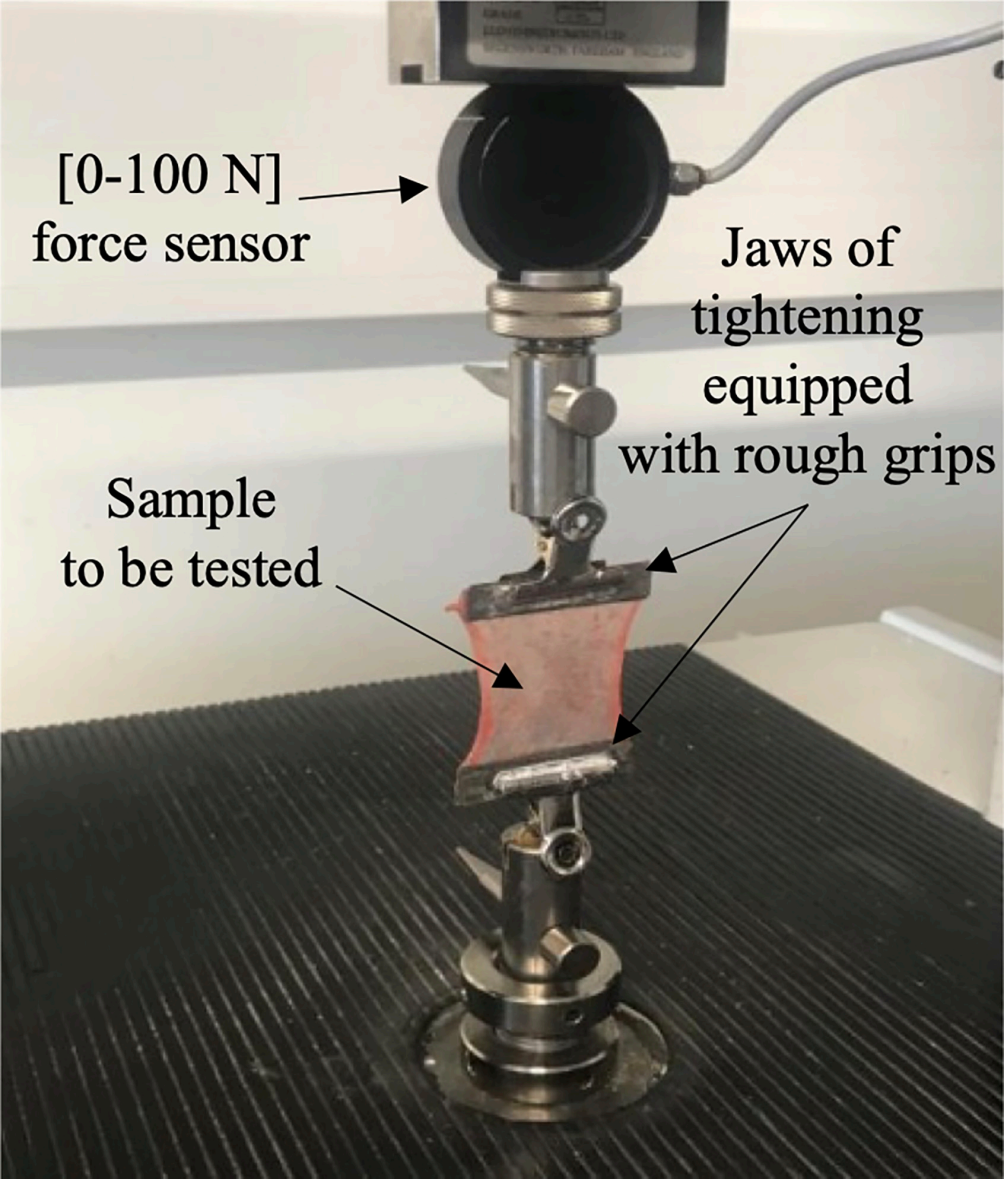
Uniaxial tensile testing.

Three to four samples 6 cm wide by 7 cm long were taken from the initial membranes. Mechanical strength tests were performed by uniaxial tensile testing with a universal testing machine (EZ20^®^, Lloyds), equipped with a force sensor whose measuring range is [0–100] Newton (N) (Fig 1). Measurements were acquired using NEXYGENplus® software. The membranes were cleaned by gentle dabbing. They were held by two jaws equipped with rough grips (pieces of silicon carbide sandpaper with a grain density of 240 per mm^2^) in order to avoid unwanted sliding of the membrane during the test.

**Fig 2:**
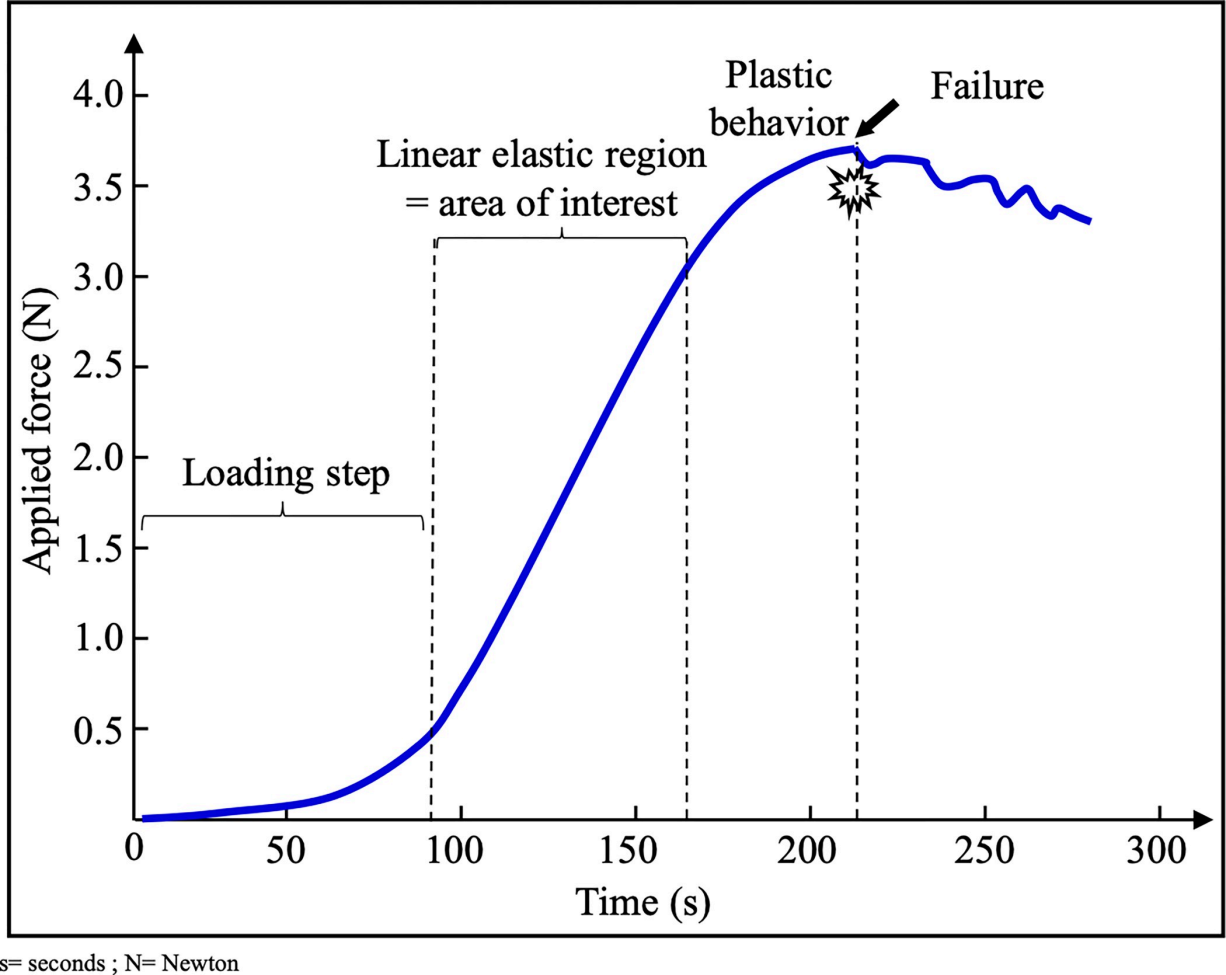
Example of an applied force/time registered curve during a uniaxial tensile test. The manipulation was started with a constant imposed stretching speed of 5 mm/minute allowing the recording of an applied force/time curve. The test was continued until the membrane ruptured. For each sample, we collected the maximum force (Fmax) before rupture (in Newtons).

The manipulation was then started with a constant imposed stretching speed of 5 mm/minute allowing the recording of an applied force/time curve (Fig 2). The test was continued until the membrane ruptured. The experiment for each membrane sample lasted a maximum of 5 minutes, and the entire manipulation did not exceed 30 minutes, to avoid drying out the membranes. For each patient, the average of the values obtained for each membrane sample tested was calculated.

**Fig 3:**
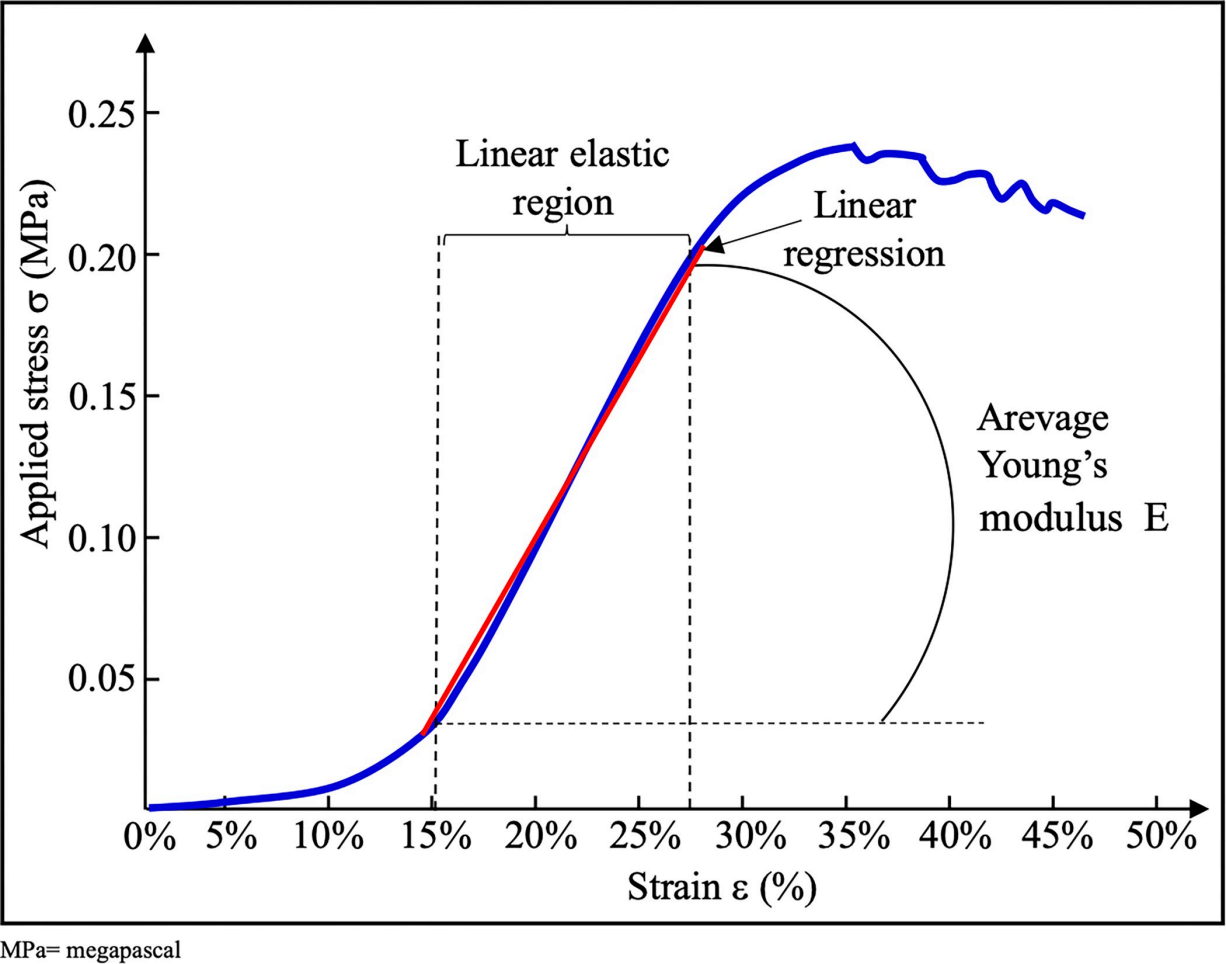
Determination of Young’s modulus from stress/strain curves. The applied stress σ corresponds to the ratio between the applied force (F) and the product of the initial length by membrane thickness. The strain ε is the ratio of the resulting length change, i.e., the final length minus the initial length, to the initial length. For each sample, we plotted force (Newton) / elongation (mm) and applied stress σ (MPa) / strain ε (%) curves on which we located the linear elastic domain. Using linear regression in Excel, we determined Young’s modulus of elasticity (E) corresponding to the slope of this domain. E, the slope of the curve in the linear domain, is evaluated by dividing the applied stress σ by the strain ε. For each sample, we collected the maximum stress before rupture σMax in megapascal (MPa).

For each sample, we plotted force (N) / elongation (mm) and applied stress σ (MPa) / strain ε (%) curves on which we located the linear elastic domain. Using linear regression in Excel, we determined Young’s modulus of elasticity (E) corresponding to the slope of this domain (Fig 3). The applied stress σ corresponds to the ratio between the applied force (F) and the product of the initial length (L) by membrane thickness (e):

$$\sigma =\frac{F}{L\;x\;e}$$

The strain ε is the ratio of the resulting length change, i.e., the final length (l) minus the initial length (L), to the initial length (L).


$$\varepsilon =\frac{l-L}{L}$$

E, the slope of the curve in the linear domain, is evaluated by dividing the applied stress σ by the strain ε.


$$E=\frac{\sigma }{\varepsilon }$$

The higher the Young’s modulus, the stiffer the fabric. The stiffness of a material is an intrinsic property independent of both the type of mechanical stress and the size of the investigated samples.

For each sample, we also collected the maximum force (Fmax) before rupture in Newtons (N) and the maximum stress before rupture σMax in megapascal (MPa). We averaged all the values obtained for each sample whose collection was technically satisfactory.

The primary endpoint of our study was σMax. The secondary endpoints were: Fmax, E, and e.

### Anatomical pathology

**Fig 4:**
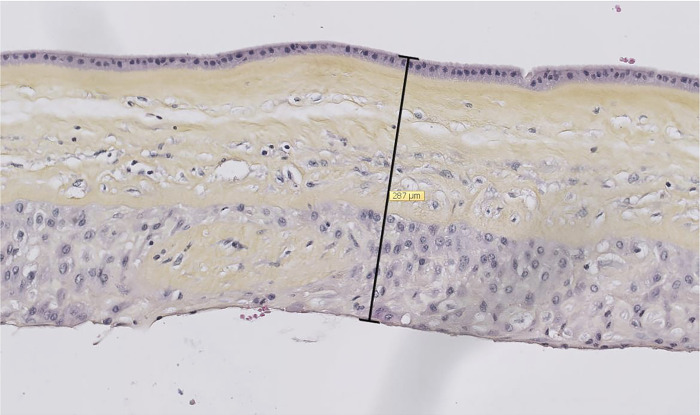
Measurement of membrane thickness on digitized image.

The membrane samples were fixed and then embedded in paraffin on the day of the cesarean. The membrane sample was then rolled up on itself and the block was cut (thickness = 3 or 4 μm) with a microtome and the section was spread on a slide. The slides obtained were then scanned (NanoZoomer 2 0 RS®, Hamamatsu) and the images processed using the NanoZoomer Digital Pathology software. For each sample, we measured the thickness of the membranes, in the area where the different membrane sheets were the most attached without edema infiltration and where the decidua was the thinnest (Fig 4).

### Statistical analyses

Quantitative variables were presented as mean ± standard deviation and qualitative variables as percentages. Comparisons of means between groups of subjects were made by Mann-Whitney or Kruskal-Wallis tests and each subject group was compared with the rest of the study population. Correlations between two quantitative variables were performed by estimating Spearman correlation coefficients. Multivariate analyses were performed by stepwise multiple linear regressions for each of the judgment criteria. The p significance level chosen for all statistical analyses was 0.05. The software used was Statistical Package for the Social Sciences (SPSS) Statistics, Version 25.0. Armonk, NY: IBM Corp.

### Ethics statement

All procedures performed in studies involving human participants were in accordance with the ethical standards of the institution and with the 1975 Helsinki Declaration and its later amendments or comparable ethical standards. CB-HME collection has been declared to the Ministry of Education and Research (DC-2011-1264) and has obtained the agreement of the ethics committee CPP Sud-Ouest Outremer IV, Limoges, France.

Written informed consent was obtained from all individual participants included in the study.

## Results

We included 33 patients, two of whom were excluded because of failed technical manipulations; 31 patients could thus be analyzed (Tables 1 and 2). The quantitative variables are presented in Table 2. All cesarean sections were performed between 37 and 39 WG +3 days, limiting the interpretation of membrane changes in relation to the advanced term of pregnancy.

**Table 1:** Qualitative characteristics of included patients.

Qualitative variables	Number	Percentage %
***Obstetrical history***		
Primiparous	9	29
Multiparous	22	71
***BMI classification***		
Underweight (<18.5)	2	6.5
Healthy weight (18.5≤BMI<25)	11	35.5
Overweight (25≤BMI<30)	5	16.1
Class 1 Obesity (30≤BMI<35)	5	16.1
Class 2 Obesity (35≤BMI<40)	5	16.1
Class 3 Obesity (BMI≥40)	3	9.7
***Medical history of prematurity***		
No	28	90.3
Yes	3	9.7
***Medical history of PPROM***		
No	30	96.8
Yes	1	3.2
***Smoking during pregnancy***		
No	19	61.3
Yes	12	38.7
***Gestational diabetes***		
No	22	71
Yes	9	29
Requiring insulin	4	44
***Geographical origin***		
Europe and North Africa	23	74.2
Sub-Saharan Africa and French West Indies	8	25.8
***Educational level***		
Out of school	5	16.1
Elementary school	2	6.5
Secondary school	10	32.3
High school	6	19.4
University education	8	25.8
***Socioeconomic level***		
Low	8	25.8
Medium	21	67.7
High	2	6.5

BMI = body mass index; PPROM = preterm premature rupture of membranes.

**Table 2:** Results for quantitative variables.

Quantitative variables	Mean	Median	Standard Derivation
Age (years)	31.1	31	5.002
BMI	28.48	29	7.728
σMax (MPa)	0.178	0.14	0.16
Fmax (N)	2.596	2.58	0.695
Young’s modulus (E)	1.06	0.96	0.532
Membrane thickness (μm)	355.9	324	99.9

BMI = body mass index; σMax = maximum stress; Fmax: Maximum force; N = Newton.

**Fig 5:**
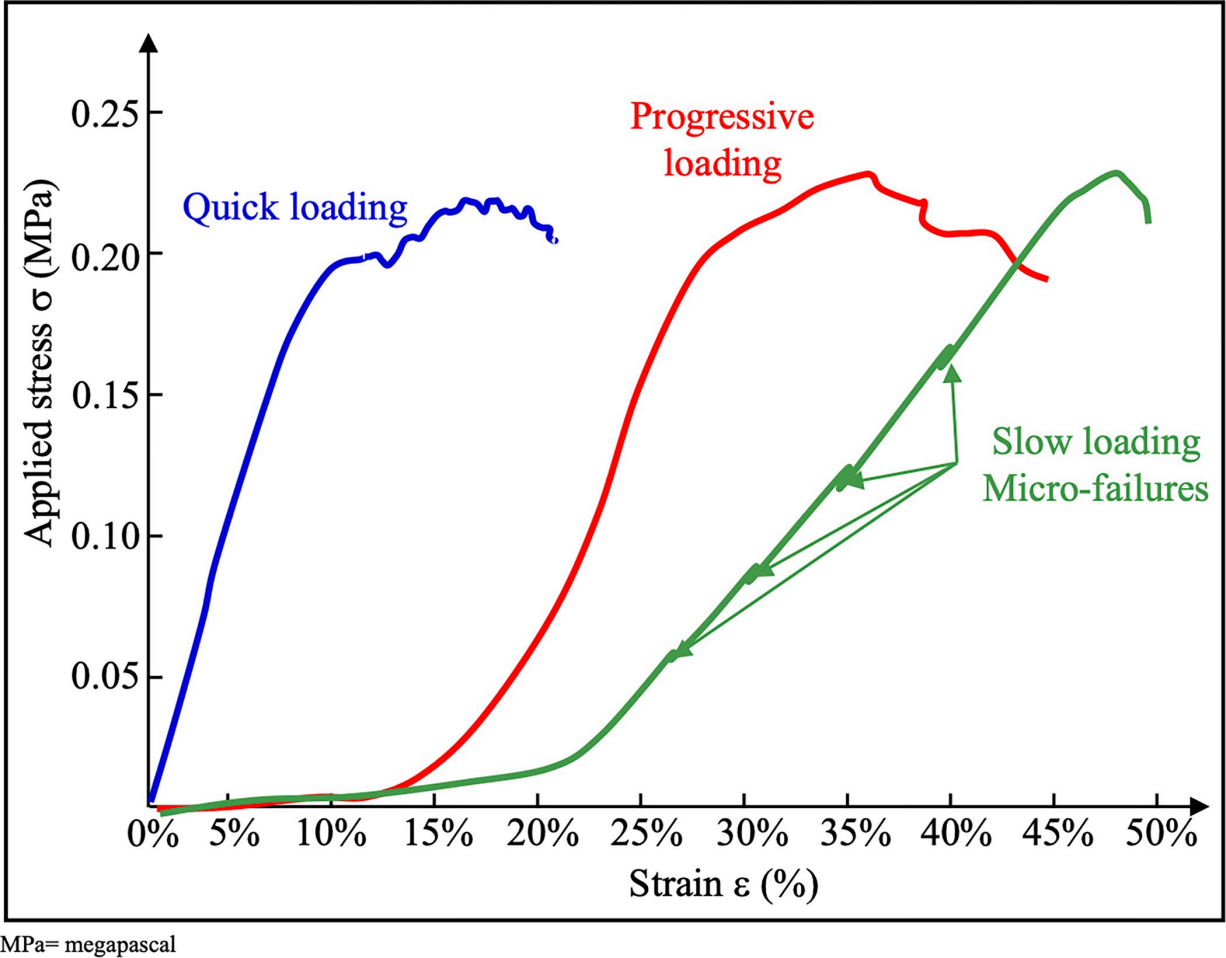
Three typical observed tensile mechanical behaviors of fetal membrane. Three types of curves were observed in the uniaxial tensile tests: Fast in blue, progressive in red and slow loading mechanical behaviors showing micro-deflections in the almost linear region in green.

The uniaxial tensile tests allowed us to observe three types of curves for the tested samples (Fig 5): quick, progressive, and slow loading mechanical behaviors exhibiting micro-failures of the tested membranes in the almost linear region were observed.

Correlation studies did not reveal any significant dependence between our primary end point σMax and E with the variables of interest studied (Table 3). We found a positive correlation between geographic origin and membrane thickness. Thus, patients of sub-Saharan or West Indian origin had thicker fetal membranes (mean 432 versus 329, p = 0.043) (Table 4) with no impact on either the Fmax applied or the σMax applied (Table 3). The membranes of primiparous patients had a significantly lower applied force before rupture compared to multiparous patients (2.16 N vs. 2.77 N, p = 0.02) and the membranes of patients with gestational diabetes, with or without insulin, had a greater applied force before rupture than patients without diabetes (3.02 N vs. 2.42 N, p = 0.033) (Table 4).

**Table 3:** Correlation between endpoints and study variables.

_Variables_^Study endpoints^	σMax (p)	Fmax (p)	Young’s modulus (p)	Thickness (p)
Age	0.434	0.864	0.283	0.357
BMI	0.987	0.096	0.685	0.447
Primiparous	0.273	**0.02**	0.273	0.915
Gravidity	0.689	0.453	0.422	0.131
Parity	0.987	0.103	0.202	0.389
Prematurity history	0.503	0.122	0.867	0.777
PPROM history	0.452	0.516	0.258	0.968
Gestational diabetes	0.654	**0.033**	0.781	0.356
Smoking	0.617	0.857	0.704	0.12
Geographical origin	0.464	0.295	0.52	**0.043**
Low socioeconomic level	0.74	0.437	0.674	0.58

BMI = body mass index; PPROM = preterm premature rupture of membranes; σMax = maximum stress; Fmax: Maximum force.

**Table 4:** Descriptive comparisons of significant correlations.

Study endpoints	Variables (n = patients)	Descriptive comparisons		
		Mean	SD	Significance (p)
Fmax (N)	Primiparous (= 9)	2.16	0.46	0.02
	Multiparous (= 22)	2.77	0.7	
	No gestational diabetes (n = 22)	2.42	0.62	0.033
	Gestational diabetes (n = 9)	3.02	0.72	
Thickness (μm)	Europe and North Africa (= 23)	329	63	0.043
	Sub-Saharan Africa and French West Indies (= 8)	432	146	

Fmax = maximum force; SD = standard deviation.

Multivariable analyses by multiple linear regressions did not reveal a significant association between the variables studied and the primary outcome σMax. We again found a significant association between thicker membranes and sub-Saharan or West Indian geographic origin (correlation coefficient B = 103.513; 95% CI [26.654; 178.372]; p = 0.01). History of prematurity was associated with a greater E and therefore with stiffer membranes (B = 0.731; 95% CI [0.117; 1.344]; p = 0.021). An increase in the number of previous deliveries was associated with a higher Fmax before rupture (B = 0.432; 95% CI [0.144; 0.720]; p = 0.005) (Table 5).

**Table 5:** Study of correlations between variables studied and judgment criteria after multiple linear regressions.

Study endpoints	Variables	Correlation coefficient B	95% CI	Significance p
σMax	All			
Fmax	Parity (quantitative)	0.432	0.141–1.06	**0.05**
Young’s modulus	Prematurity history	0.731	0.117–1.344	**0.021**
Thickness	Sub-Saharan Africa or French West Indies	102.513	26.654–178.372	**0.01**

σMax = maximum stress; Fmax = maximum force; CI = confidence interval.

We confirmed the expected positive correlation between σMax and Fmax (r = 0.586; p = 0.001) and found significantly negative correlations between σMax and thickness (r = -0.460; p = 0.009) as well as between E and thickness (r = -0.376; p = 0.037), meaning that thicker fetal membranes tend to be less stiff and have less resistance to a lower maximum applicable stress. We did not find a significant correlation between E and Fmax (r = 0.043; p = 0.817) or σMax (r = 0.256; p = 0.165), so we did not find a relationship between membrane stiffness and strength.

## Discussion

### Main findings

PPROM is a frequent obstetrical event whose pathophysiology is still poorly understood although infection and inflammation appear to be the main causes [11,25,26]. The immune function of pregnant women is impaired, facilitating the upward invasion of pathogens to nearby fetal membranes. This phenomenon triggers the production of various hydrolytic enzymes acting on the extracellular matrix of fetal membranes, initiating the hydrolysis process. Fetal membrane fragility can thus occur at the same time as a decrease in local surface tension, leading to PPROM [11]. Our objective was to provide new insights into the possible links between mechanical fragility of membranes and risk factors for PPROM outside an infectious context. Although no relationship was found with our primary endpoint, analysis of the secondary endpoints is consistent with some of the literature.

In our study, the σMax applied before rupture was on average 0.178 MPa. A meta-analysis that tested the whole membrane (without separation of the amnion and chorion), with different test methods, found a lower value of around 0.09 MPa for a gestational age >37 WG [21]. However, the studies analyzed in this publication used membranes of fetuses born vaginally and therefore already subject to the biochemical changes of labor and to prior rupture. More recently, Verbruggen et al. [22] modeled a σMax of 0.145 MPa at 40 WG, which is close to the figures observed in our study. However, they noted that this value, applied to the chorioamniotic membrane as a whole, would in fact correspond to a stress applied at 0.989 MPa if the amnion was taken separately, which corroborates the fact that rupture starts with the chorion.

In our study, the Fmax before rupture was on average 2.6 N. Oxlund et al. demonstrated a lower Fmax of 0.95 N in uniaxial tensile testing of term vaginal fetal membranes [23,24]. They then replicated their experience with membranes from fetuses born at term by scheduled cesarean section and observed a higher Fmax at 1.39 N [24]. These lower figures could be explained by the fact that this team performed the traction tests by immersing the membrane in a Ringer lactate solution at room temperature (pH 7.4). We did not wish to immerse or rinse the membrane in saline because we had noticed, during our preliminary tests, that the chorioamniotic sheets tended to separate; the separation of these 2 sheets contributes to the weakening of the membranes [27,28].

We found a Young’s modulus E of 1.06 on average, which is difficult to compare with literature values because of the heterogeneity of the results [21]. Helmig et al., in their uniaxial tensile test, showed a Young’s modulus of 7.1 MPa for the 7 fetal membranes obtained by programmed cesarean section at term, and tested without separating the chorion and the amnion [24]. The heterogeneity of the results of the different studies can be explained by the viscoelastic nature of the membranes [29,30]. The particularity of a viscoelastic material is that its mechanical behavior law depends on time, and therefore on the speed of solicitation. It can involve a delay between the application of a force on the membrane and the attainment of its final deformation equilibrium in response to this force. This viscoelastic character is clearly visible in the modeling of our 3 types of stress/strain curves (Fig 5). Indeed, the membranes had a strong capacity to shrink on themselves at the time of sample collection, which could lead to slightly different sample sizes in the end. The 45 mm length of fabric between the 2 jaws at the beginning of the test, which was identical in all our experiments, could lead to very tense membranes from the start, with immediate loading and a curve starting immediately in the linear domain (blue curve). On the other hand, a less tense membrane at the beginning of the test (without preloading) took more time to reach its equilibrium point and to be loaded (green curve).

Thickness measurement is necessary to evaluate the stress. The thickness of our membranes was 355.9 μm on average. This measurement was made on histological sections of fixed tissue; the thickness thus measured was not necessarily identical to that of the tested membranes. Moreover, the membranes could present variations in thickness in the same sample. To limit this bias, we measured the most reproducible area, with the least amount of detachment between the chorion and the amnion or where the decidua was the least thick. Depending on the authors and the techniques used, the literature reports figures ranging from 191 to 489 μm [23,24,31,32].

We found a lower Fmax in our primiparous patients and an increase in Fmax in the case of previous deliveries, which suggests a lower resistance of fetal membranes in primiparous patients. However, this result was not found for σMax, which could be explained by the fact that our numbers were too small to show a significant difference or by the bias in the collection of the thickness of the membranes involved in the calculation of σMax. We found a greater Fmax in patients with gestational diabetes. This result seems to us difficult to explain pathophysiologically and is at odds with the study of Bouvier et al. who described it as a risk factor of PPROM [10]. It is possible that in the long term the effect on the mechanics of the membranes is different from before 37 WG.

Our results show that as the thickness of the membranes increases, the stiffness (Young’s modulus E) and the σMax before rupture decrease. This finding could be explained by a different distribution of collagen and by a greater infiltration of edema in the thickest tissues, but also by the fact that cleavage between the amnion and the chorion increases the thickness of the membrane but also weakens it. We found a significantly increased membrane thickness in patients of sub-Saharan and West Indian origin without modification of σMax and Fmax, making the interpretation of this parameter difficult. African ethnicity has been described as a risk factor for PPROM in US studies, but an increased risk of preterm birth was found only in US-born black mothers and not in African immigrant patients [33,34]. Ethnicity is a controversial risk factor for preterm birth [35]. The differences observed between black mothers of American origin and black mothers of African origin could be explained by the fact that the genetic elements that determine the black skin phenotype are not the same as those responsible for obstetrical complications such as PPROM [36]. This susceptibility to PPROM in Afro-American mothers could be explained by the occurrence of mutations in several genes encoding proteins involved in the attenuation of the innate immune response or host protection against microbial infections [37]. Modi et al. identified nonsense mutations in newborns of Afro-American patients in the *DEFB1* and *MBL2* genes that encode antimicrobial proteins and defend fetal membranes against infectious agents [38]. Wang et al. also worked on the *SERPINH1* gene, which codes for a protein involved in collagen synthesis. This gene is thought to be more frequently mutated in Afro-American patients and predisposes to PPROM [39].

### Clinical implications

This study provides further insight into the pathogenesis of PPROM. For early identification, monitoring, treatment, and avoidance of negative consequences, it is essential to identify risk factors and to understand the various mechanisms that cause preterm labor. Moreover, determining experimentally the tensile law of behavior of the membranes (stress/strain curves) can be valuable in the objective of feeding reliable data to the software of numerical modeling used to predict the mechanical behavior of certain parts of the human body.

### Research implications

While we were unable to establish a link between some of the risk factors for PPROM classically described in the literature and mechanical fragility of the membranes, biochemical analysis (collagen, elastin) by multiphoton microscopy of fetal membranes according to PPROM risk factors could help us to understand the pathogenesis of these elements, and would be an interesting complement to our study of the mechanical behavior of these membranes. The use of ultrasound elastography could also be an interesting line of research to study the mechanical behavior and elasticity of fetal membranes in patients at risk of PPROM.

### Strengths and limitations

Four methods of mechanical testing of fetal membranes are described [21]: uniaxial tensile, biaxial inflation, biaxial puncture, and planar biaxial tensile. The most popular testing method in recent years has been the biaxial puncture test (probe applied at a constant velocity to the center of the membrane held taut by a circular ring until rupture), which is thought to more realistically simulate intrauterine physiology [40–44], with pressure applied on the zone of cervical weakness by amniotic fluid and later by fetal movement [29,30]. However, the very complicated geometry of this type of test makes it difficult to analyze the data and many times authors could not calculate the stress σ and Young’s modulus. Although debatable, we chose, for our innovative study, uniaxial traction for its simplicity of implementation, for the availability of the equipment and for its geographical proximity to our sampling center.

We performed our tests on membranes of full-term fetuses so as to be able to test membranes not modified by labor and not degraded by vaginal delivery. These membranes may have a different behavior from membranes of preterm fetuses, thus representing a limitation to our results. Nevertheless, most markers of oxidative stress, senescence and inflammation of fetal membranes are similar in PPROM and normal term births [45]. At term, ZAM, show altered morphology [46,47]. The study of membrane thickness focused on an area of the membranes further away from the ZAM, and closer to the placenta, which introduces a bias into our study, as these areas are functionally and biologically different. We chose to perform our tests on the ZAM because this is the zone where PPROM occurs most frequently. The variation in the term at which the cesarean section was performed seems to us to have played a minimal role in the interpretation of the results, insofar as the surgery was always performed before 39 WG and 3 days.

We had no previous data with which to estimate a specific sample size for our study, which is why we chose to include consecutive participants on a prospective basis. As our main task was to estimate correlation coefficients, we chose to focus on a 6-month period, which enabled us to include more than 30 participants, but may have limited the power of some secondary analyses.

To our knowledge, and although limited by our small sample size, ours is the only study that has investigated the impact of clinical risk factors for PPROM on the mechanical strength of fetal membranes. We believe that this work is an interesting preamble to a larger study in which the different membrane zones could be tested and compared, but also where the effect of the association of several clinical risk factors could be investigated.

## Conclusion

Studying the pathophysiology of PPROM is an important issue that will enable better management of patients at risk. We have demonstrated a greater mechanical fragility of the membranes in primiparous patients, which may explain the greater frequency of PPROM in these patients. We did not find any impact on the resistance of the fetal membranes of maternal age, BMI, smoking during pregnancy, European or sub-Saharan geographical origin, or socioeconomic level. We found a greater fragility of the membranes, and a decrease in their rigidity, when their thickness increased.
